# Bioinformatics analyses on the immune status of renal transplant patients, a systemic research of renal transplantation

**DOI:** 10.1186/s12920-020-0673-6

**Published:** 2020-02-11

**Authors:** Mei Meng, Weitao Zhang, Qunye Tang, Baixue Yu, Tingting Li, Ruiming Rong, Tongyu Zhu, Ming Xu, Yi Shi

**Affiliations:** 1 Shanghai Key Laboratory of Organ Transplantation, Shanghai, China; 2 Institute of Clinical Science, Zhongshan Hospital Fudan University, Fenglin Road 180, Shanghai, PRC CN-200032 China; 3 Department of Urology, Zhongshan Hospital Fudan University, Fenglin Road 180, Shanghai, PRC CN-200032 China

**Keywords:** Bioinformatics, Kidney transplantation, Immune regulation

## Abstract

**Background:**

Kidney transplantation is the most effective treatment for end-stage renal disease. Allograft rejections severely affect survivals of allograft kidneys and recipients.

**Methods:**

Using bioinformatics approaches, the present study was designed to investigate immune status in renal transplant recipients. Fifteen datasets from Gene Expression Omnibus (GEO) were collected and analysed. Analysis of gene enrichment and protein-protein interactions were also used.

**Results:**

There were 40 differentially expressed genes (DEGs) identified in chronic rejection group when compared with stable recipients, which were enriched in allograft rejection module. There were 135 DEGs identified in acute rejection patients, compared with stable recipients, in which most genes were enriched in allograft rejection and immune deficiency. There were 288 DEGs identified in stable recipients when compared to healthy subjects. Most genes were related to chemokine signalling pathway. In integrated comparisons, expressions of MHC molecules and immunoglobulins were increased in both acute and chronic rejection; expressions of *LILRB* and *MAP 4 K1* were increased in acute rejection patients, but not in stable recipients. There were no overlapping DEGs in blood samples of transplant recipients.

**Conclusion:**

By performing bioinformatics analysis on the immune status of kidney transplant patients, the present study reports several DEGs in the renal biopsy of transplant recipients, which are requested to be validated in clinical practice.

## Introduction

Kidney transplantation is the most effective treatment for end-stage renal disease. However, acute and chronic graft rejections affect survivals of allograft kidneys and transplant patients [1, 2]. Acute rejection is characterised by a quick loss of renal function, whereas chronic rejection presents gradual development of renal failure. Pathological diagnosis is the best approach to assess disease classification and differentiate complications. However, many cases are diagnostically difficult since disease processes share nonspecific mechanisms, including innate immunity [3, 4], inflammation [5–7], and microcirculation remodelling [8].

Mechanisms and key regulators underlying the development of allograft rejection are complicated. Increased presences of major histocompatibility complex (MHC, also known as human leukocyte antigen HLA) are found in allografts, from both acute and chronic rejection patients, demonstrating that MHC upregulation is the crucial issue in the allograft immune responses. Meanwhile, the immune system from recipients targets foreign MHC proteins and triggers allograft immune responses. T lymphocytes take a significant part in the process of acute rejection, while B lymphocytes are more critical in the development of transplant tolerance and chronic rejection [9, 10]. Thus, it is crucial to understand the immune status and the involvement of lymphocytes in transplant recipients.

In the last decade, gene microarray has been extensively used in transplant immunology [11]. Data from renal biopsies and liquid biopsies provide potential molecular signatures and precision assessments in the immune status of allograft recipients [12]. It is reported that upregulated genes in renal biopsies from acute rejection patients are involved in immune and inflammatory responses, whereas downregulated genes are more involved in different categories of cellular metabolism [13]. In recipients with stable kidney function, DEGs are classified into cell growth, protein metabolism as well as transcription factors, indicating subclinical immune responses [14]. Compared with stable recipients, acute rejection patients present complement activation and lower expressions of serpin family protein in plasma, indicating increased systemic inflammation and impaired vascular permeability [7]. In peripheral blood samples, increased type I interferon signaling represents a molecular signature in chronic antibody-mediated rejection [15]. Of note, underlying molecular regulations in inflammation and immune responses, both innate and adaptive response, are months before histologic lesions appear [16]. Thus, to better understand changes of immune state and underlying molecular regulation in transplant recipients, the present study recruited 15 datasets of renal transplant recipients from GEO. Analysis of gene enrichment and protein-protein interactions were also performed to identify potential regulators in the progress of allograft rejection.

## Method

### Microarray datasets and groups

**Fig. 1:**
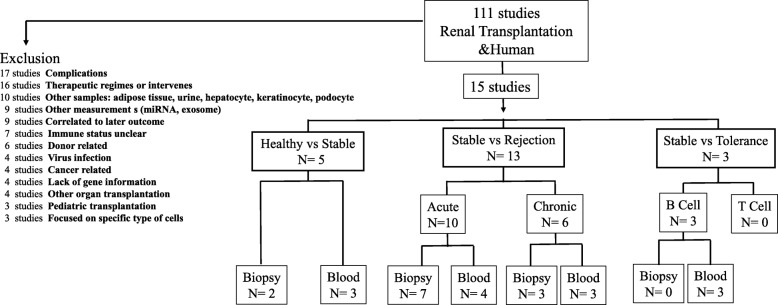
A diagram of the dataset recruitment workflow

There were 111 datasets regarding human kidney transplantation in the GEO database (http://www.ncbi.nlm.nih.gov/pubmed/GEO). The present study recruited 15 datasets of renal transplant patients with stable condition, acute rejection, chronic rejection or immune tolerance [7, 13, 15–26]. Studies involving acute kidney injury, pediatric transplantation, or different therapeutic regimes on immune response were excluded (Fig. 1, Additional file 1: Table S1). All 15 studies were carried out in North American [7, 13, 16, 18–21, 23–26] or Europe [15, 17, 22]. Most participants in the studies were Caucasian, while a few subjects were African-American, Asian or American Indian.

In the present study, five datasets were examined immune statuses of healthy subjects and stable recipients, two for renal biopsies [13, 17] and three for blood samples [13, 22, 23]. Ten studies were focused on acute rejection when compared with stable recipients, seven for renal biopsies [13, 16–21] and four for blood samples [7, 13, 24, 25]. Six studies were pooled to study immune responses in chronic rejection when compared with stable recipients, three for renal biopsies [17, 19, 20] and three for blood samples [15, 22]. Also, there were three sets examined the immune status in transplant tolerant recipients, focusing on the gene signature of peripheral B lymphocytes [22, 23, 26] (Fig. 1, Tables 1 and 2).

**Table 1:** GEO datasets of renal biopsies from kidney transplant recipients

	Data Sets	Sample size		Up-regulated	Down-regulated
Stable vs Healthy	GSE9493 [17]	Stable	21	1422	1742
		Healthy	15		
	GSE1563 [13]	Stable	10	1159	1149
		Healthy	9		
Acute vs Stable	GSE9493 [17]	Acute	10	3	3
		Stable	21		
	GSE1563 [13]	Acute	6	1178	1247
		Stable	9		
	GSE50058 [18]	Acute	43	1945	2328
		Stable	58		
	GSE25902 [16]	Acute	24	6964	6915
		Stable	96		
	GSE36059 [19]	Acute	35	1422	965
		Stable	281		
	GSE98320 [20]	Acute	81	1042	684
		Stable	774		
	GSE106675 [21]	Acute	10	2107	2022
		Stable	6		
Chronic vs Stable	GSE9493 [17]	Chronic	25	1377	1227
		Stable	21		
	GSE36059 [19]	Chronic	65	236	23
		Stable	281		
	GSE98320 [20]	Chronic	326	271	5
		Stable	774

**Table 2:** GEO datasets of blood samples from kidney transplant recipients

	Data Sets	Sample Size		Up-regulated	Down-regulated
Stable vs Healthy	GSE1563 [13]	Stable	9	915	947
		Healthy	8		
	GSE47755 [22]	Stable	380	26	8
		Healthy	16		
	GSE22229 [23]	Stable	27	173	295
		Healthy	12		
Acute vs Stable	GSE1563 [13]	Acute	6	49	57
		Stable	9		
	GSE14346 [24]	Acute	38	1015	1563
		Stable	37		
	GSE15296 [25]	Acute	51	591	707
		Stable	24		
	GSE46474 [7]	Acute	20	15	4
		Stable	20		
Chronic vs Stable	GSE47755 [22]	Chronic	78	2	0
		Stable	380		
	GSE51675 [15]	Chronic	10	2	0
		Stable	8		
	GSE64261 [15]	Chronic	5	0	0
		Stable	5		
Tolerance vs Stable	GSE47755 [22]	Tolerance	54	1	3
		Stable	380		
	GSE22229 [23]	Tolerance	19	72	8
		Stable	27		
	GSE66612 [26]	Tolerance	81	51	61
		Stable	77		

There were no animal or human subjects involved in the present study.

### Data processing and analysis

The 15 datasets were downloaded from the GEO database and analyzed separately. The preprocessing of the microarray dataset with raw data was performed by using the Affy package in the R environment (version 3.4.2, https://www.R-project.org). For background correction, normalization, and differentially expressed genes (DEGs) screening, *limma* and *impute* packages were used in the present study [27]. DEGs, both upregulated and downregulated, were defined when absolute log2 FC was higher than 0.5 and an adjusted *p*-value was less than 0.05. To correct multiple hypotheses, Benjamini-Hochberg false discovery rate correction was used to adjust p-value in the present study. Annotation files for different microarray platforms are downloaded from the GEO database as well. STRING, an online tool, was used to explore protein-protein interactions [28]. ClueGO, a plug-in in Cytoscape 3.6.1, was used to group functional proteins and visualise their biological terms [29].

The overlapping genes in integrated comparisons were visualised by using the Venn package in R. Changes of genes in the intersection were normalised with the controls in the same dataset, and presented as fold changes. The statistical analysis between two groups was done by two-tailed student t-test. Differences were considered to be statistically significant when *p*-value was less than 0.05.

## Results

### Gene profiling of renal biopsies from transplant recipients

#### Chronic rejection vs stable recipients

**Fig. 2:**
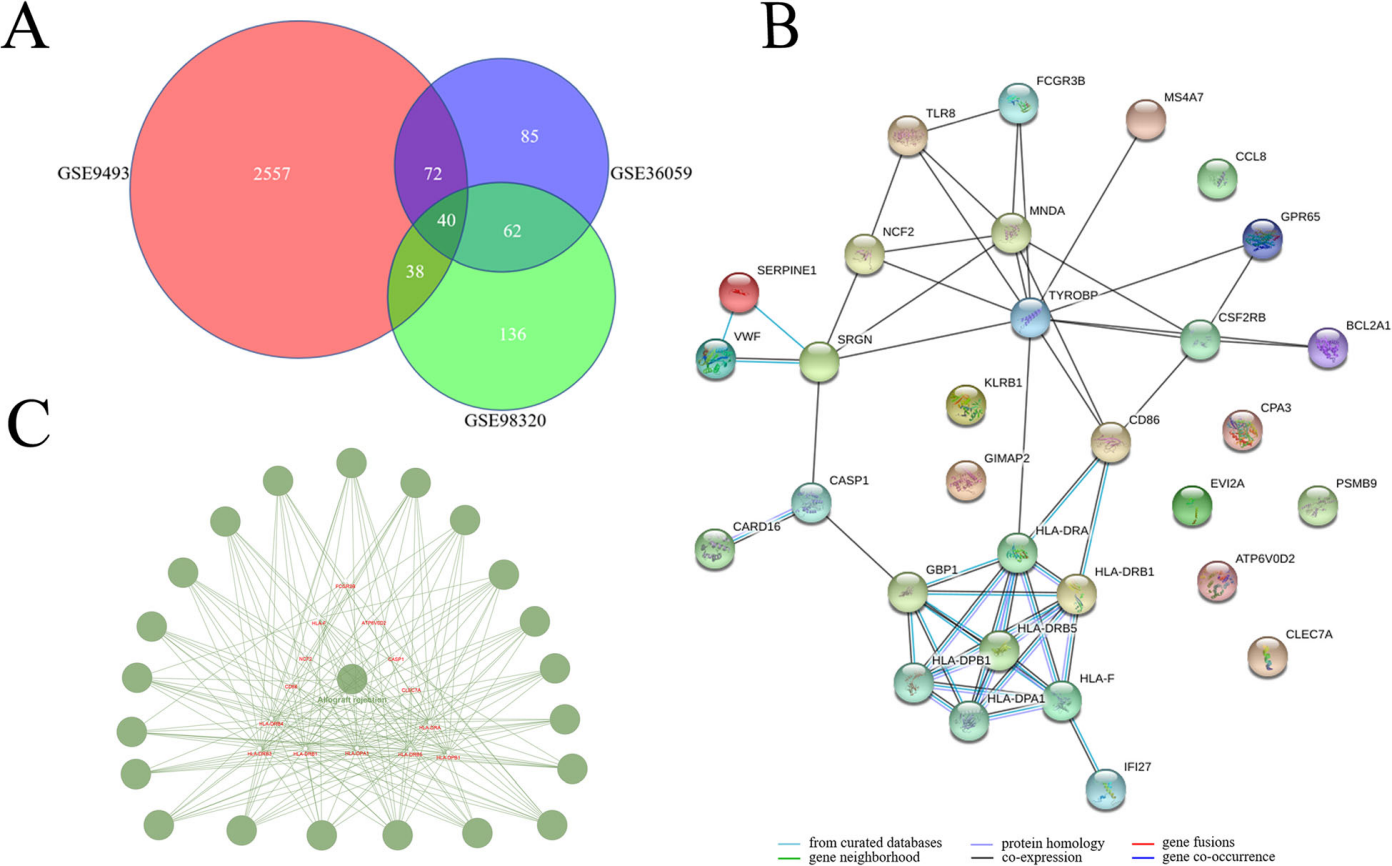
Analysis DEGs in renal biopsies, comparing chronic rejection patients with stable recipients. **a** Intersection analysis of DEGs by Venn diagram; (**b**) Protein-protein interactions were visualized by STRING. Each node represents a gene, and each line refers to an interaction; (**c**) Enrichment of biological functions in the DEGs. The node size represents the number of DEGs, and the node colour represents a module in the enrichment classification

Three datasets were comparing renal biopsies of chronic rejection patients with stable recipients. A total of 40 DEGs were found in chronic rejection patients (Fig. 2a). All the genes were enriched in the allograft rejection module (Fig. 2b and 2 c).

#### Acute rejection vs stable recipients

**Fig. 3:**
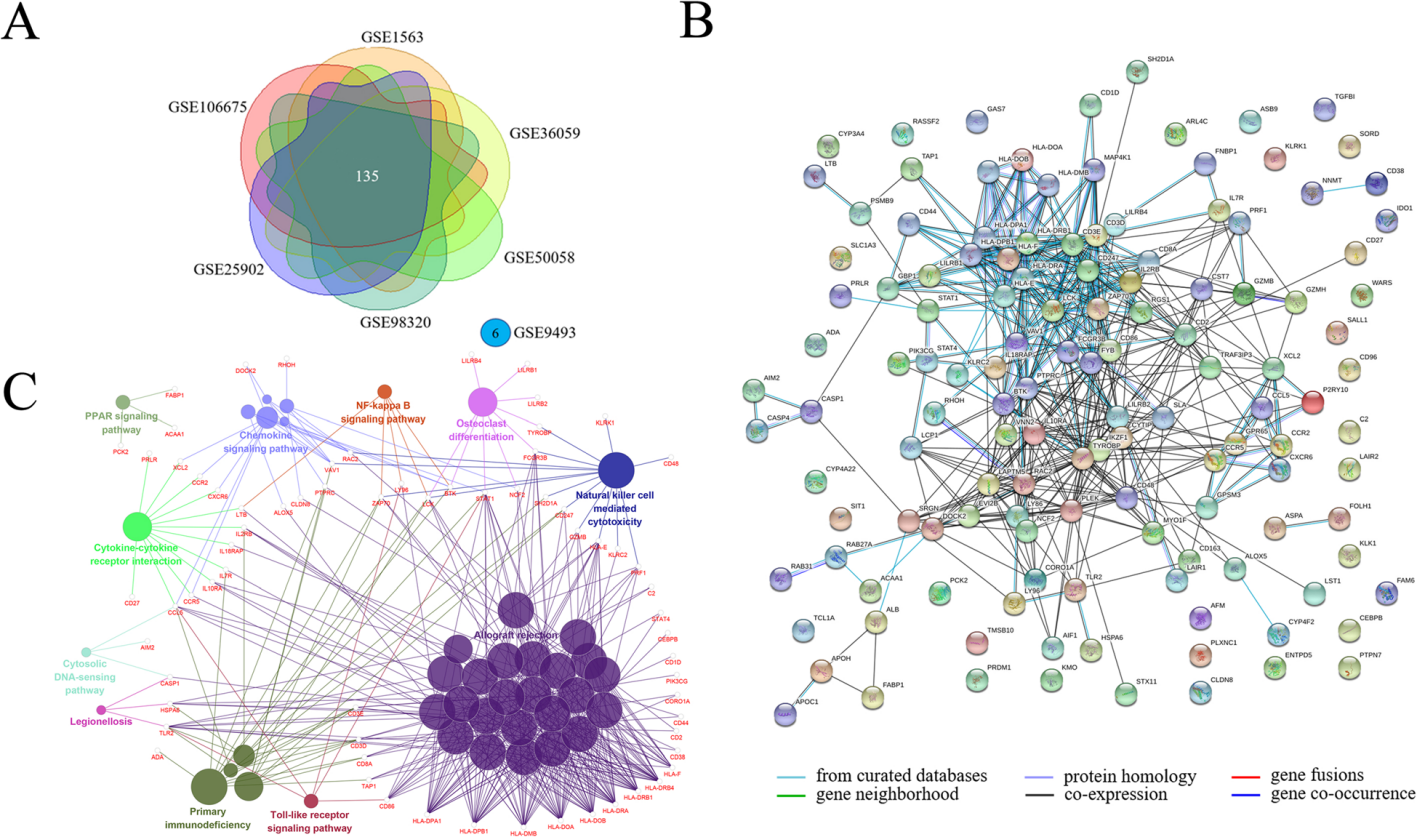
Analysis DEGs in renal biopsies, comparing acute rejection patients with stable recipients. **a** Intersection analysis of DEGs by Venn diagram; (**b**) Protein-protein interactions were visualized by STRING. Each node represents a gene, and each line refers to an interaction; (**c**) Enrichment of biological functions in the DEGS. The node size represents the number of DEGs, and the node colour represents a module in the enrichment classification

Seven datasets were comparing renal biopsies from acute rejection patients with stable subjects. After removing GSE9493 since only six DEGs were found, a total of 135 DEGs were found in common (Fig. 3a). ClueGO showed that 58.4% of genes were enriched in allograft rejection module, 12.2% in the chemokine signalling pathway module, and 9.76% in primary immunodeficiency module. Other modules included nuclear factor kappa B (NF-κB) signalling pathway, Toll-like receptor (TLR) signalling, and peroxisome proliferator-activated receptors signalling pathway (Fig. 3b and 3 c).

#### Stable vs healthy subjects

**Fig. 4:**
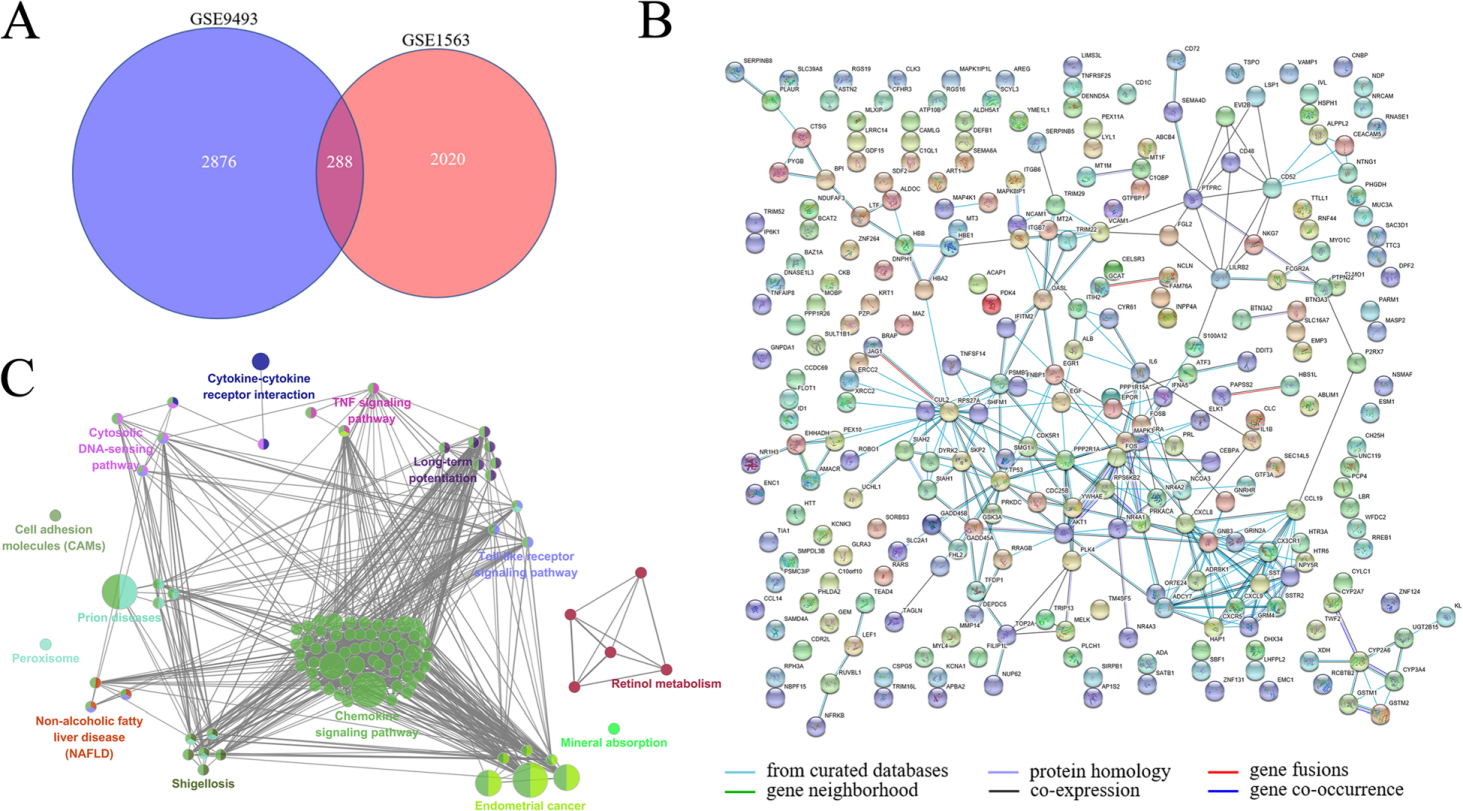
Analysis of DEGs in renal biopsies, comparing stable recipients with healthy subjects. **a** Intersection analysis of DEGs by Venn diagram; (**b**) Protein-protein interactions were visualized by STRING. Each node represents a gene, and each line refers to an interaction; (**c**) Enrichment of biological functions in the DEGs. The node size represents the number of DEGs, and the node colour represents a module in the enrichment classification

Two datasets were comparing renal biopsies of healthy subjects and stable recipients. A total of 288 DEGs were found in stable recipients compared with healthy subjects (Fig. 4a). ClueGO showed that 61.7% of genes were enriched in chemokine signalling pathway module; other modules included prion diseases, TLR signalling, endometrial cancer, long-term potentiation, and shigellosis. (Fig. 4b and c).

#### Progressive changes in integrated comparisons

**Fig. 5:**
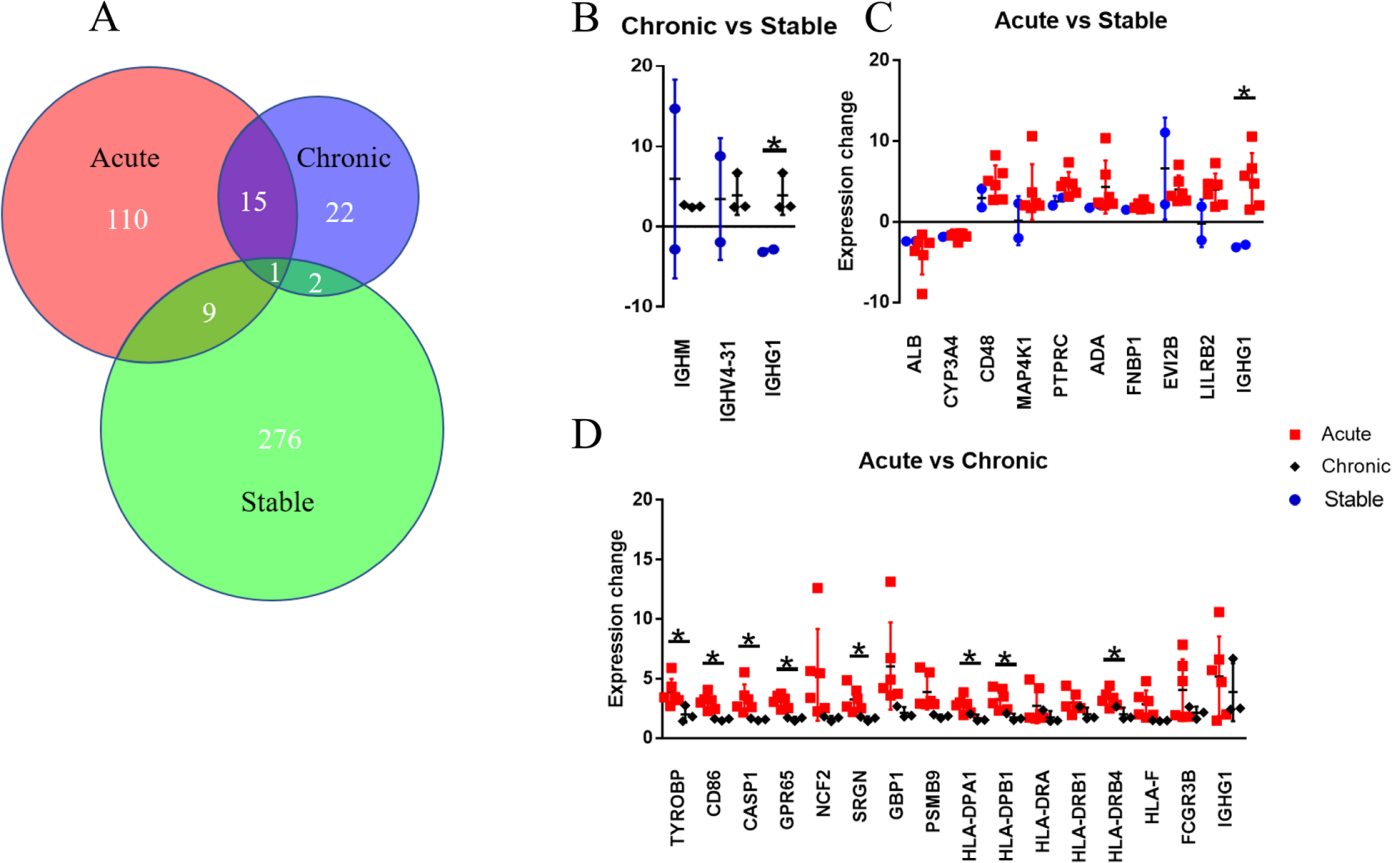
**a** Integrated analysis of DEGs from renal biopsies of stable, acute rejection and chronic rejection groups. **b** Scatter plots of three DEGs identified in the comparison of chronic rejection patients and stable recipients. Gene changes are normalised with controls in the same dataset and presented as fold changes individually. Data are shown as means ± standard error of the mean; 3 sets of chronic rejection and 2 sets of stable groups. **c** Scatter plots of 10 DEGs identified in the comparison of stable and acute rejection patients. Gene changes are normalised with controls in the same dataset and presented as fold changes individually. Data are shown as means ± standard error of the mean; 2 sets of stable subjects and 6 set of acute rejection. **d** Scatter plots of 16 DEGs identified in the combined comparison of acute rejection and chronic rejection group. Gene changes are normalised with controls in the same dataset and presented as fold changes individually. Data are shown as means ± standard error of the mean; 6 sets of acute rejection and 3 sets of chronic rejection. * *P* < 0.05 vs acute rejection group

To understand changes of immune status in transplant patients, DEGs identified above were further analysed in combined comparisons (Fig. 5a).

There were three DEGs found in both stable and chronic rejection groups. Immunoglobulin heavy constant Mu (*IGHM*), immunoglobulin heavy variable 4–31 (*IGHV4–31*), and immunoglobulin heavy constant gamma of 1 (*IGHG1*) were upregulated in chronic rejection patients, but not in stable recipients. (Fig. 5 B, Additional file 1: Table S2).

There were 10 DEGs found in both stable and acute rejection groups. Among them, albumin (*ALB*) and cytochrome P450 3A4 (*CYP3A4*) were downregulated, while cluster of differentiation 48 (*CD48*), protein tyrosine phosphatase receptor type C (*PTPRC*, also known as *CD45*), adenosine deaminase (*ADA*), and formin binding protein 1 (*FNBP1*), ecotropic viral integration site 2B (*EVI2B*) were upregulated. Expressions of mitogen-activated protein kinase kinase kinase kinase 1 (*MAP 4 K1*), leukocyte immunoglobulin-like receptor B2 (*LILRB2*), and *IGHG1* were increased in acute rejection patients, but not in stable transplant recipients (Fig. 5c, Additional file 1: Table S3).

There were 16 upregulated genes found in both acute and chronic rejection comparisons, including major histocompatibility complex class I (*HLA-F*) and II (*HLA-DPA1, DPB1, HLA-DRA, DRB1,* and *DRB4*), *IGHG1*, Fc fragment of IgG receptor IIIb (*FCGR3B*, also known as *CD16b*), *CD86*, proteasome subunit beta 9 (*PSMB9*), guanylate binding protein 1(*GBP1*), serglycin (*SRGN*), neutrophil cytosolic factor 2 (*NCF2*), G protein-coupled receptor 65 (*GPR65*), caspase 1 (*CASP1*), and TYRO protein tyrosine kinase binding protein (*TYROBP*).

Of note, expressions of *HLA-DPA1, DPB1, DRB4, CD86, GPR65, CASP1, TYROBP,* and *SRGN* were significantly higher in the acute rejection group than those in the chronic one (Fig. 5d, Additional file 1: Table S4).

### Gene profiling of peripheral blood lymphocytes from kidney transplant recipients

**Fig. 6:**
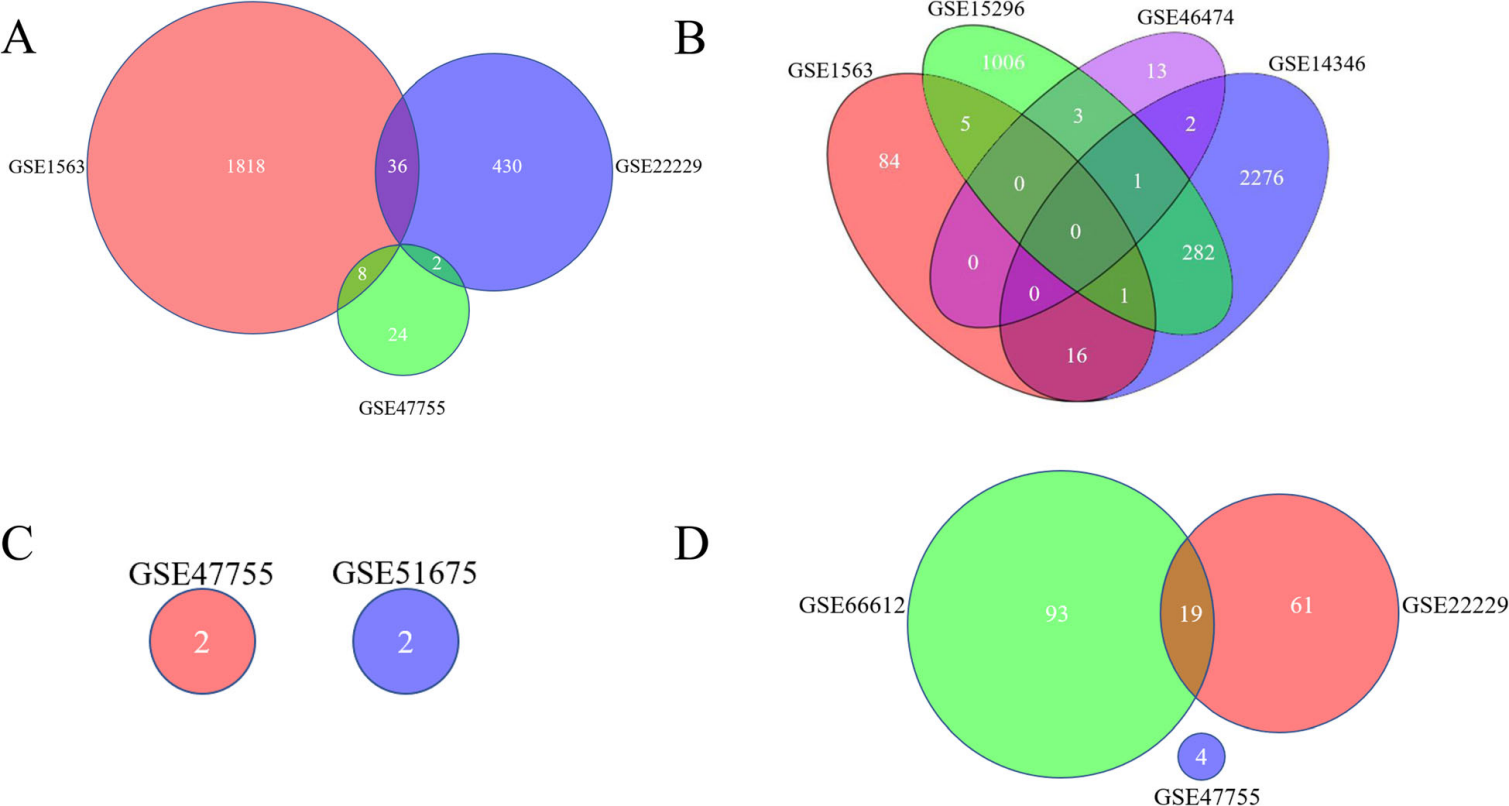
Analysis of DEGs in the blood samples. (**a**) Combined comparison of stable recipients with healthy subjects. (**b**) Combined comparison of acute rejection patients with stable subjects. (**c**) Combined comparison of chronic rejection patients with stable recipients (**d**) Combined comparison of tolerance recipients with stable recipients

Likewise, comparisons of blood samples of transplant recipients were performed. There were no DEGs overlapped in combined comparisons (Fig. 6a-d).

## Discussion

The present study performed systematic research on immune status in renal transplant recipients. Using bioinformatics approaches, molecular patterns were analysed in renal biopsies and peripheral blood lymphocytes of transplant recipients. The main findings include A) Upregulation of *MHC* presences is critical in initiating immune responses in both acute and chronic rejection. B) Increased expressions of *LILRB* and *MAP 4 K1* are potential checkpoints for the occurrence of acute rejection. C) Gene profiles of peripheral blood lymphocytes are not in line with those of renal biopsies.

Increased expressions of *MHC* molecules were found in both acute and chronic rejection, confirming the critical role of MHC in allograft immune responses. MHC II proteins, including HLA-DRA, −DPA, −DPB, −DQA and DQB, confer heterodimeric protein receptors in cell membrane. Allograft recipients with donor-specific HLA-DP antibodies before the surgery suffer much severer antibody-mediated rejections than those without [30, 31]. PSMB9 is known as 20S proteasome subunit beta-1i. When cells challenged with interferon-gamma, PSMB9 constitutes immunoproteasome to process MHC I molecules [32, 33]. The upregulation of PSMB9 has been reported in zero-hour [34, 35] and 6-month [36] renal biopsies as a potential candidate to predict acute and chronic graft nephropathy. CD86 offers costimulatory signals for T cell activation. The upregulation of CD86, together with the increased expressions of HLA primed the recruitment of T lymphocytes, revealing a fundamental mechanism of allograft rejections. Furthermore, gene expressions of *HLA-DPA1, DPB1, DRB4,* and *CD86* were higher in the acute rejection than those in the chronic rejection, indicating that acute rejection induces stronger allograft immune responses.

Besides CD86 which is essential for T cell activation, other cell surface molecules were also identified in renal biopsies. Both *CD45* (also known as PTPRC) and *CD48* expressions are upregulated in rejection patients [37]. CD45 is the common leukocyte antigen, which is critical in regulating T- and B-cell antigen receptor signalling. CD48 is a member of the lymphocyte activation signalling family. By interacting with other ligands, CD48 participates in graft rejection [38]. Combined treatment of anti-CD2 and -CD48 in vivo significantly improves mouse cardiac allograft survival, while administration of either antibody alone has little effects [39]. Combined therapy of anti-CD48, anti-lymphocyte function-associated antigen 1 and fingolimod (a sphingosine 1-phosphate receptor modulator for multiple sclerosis treatment) keeps embryonic pig pancreas function in diabetic mice [40]. CD16 is required for antibody-dependent cell-mediated cytotoxicity processes in human monocytes [41].

Of note, IGHG1 was the only DEG found in all the renal biopsies, which was upregulated in both acute and chronic rejection patients but downregulated in stable recipients. Together with the other two genes encoding immunoglobulin heavy chains, *IGHV4–31* and *IGHM*, it indicates that B lymphocytes are activated and differentiated when challenged with antigen in the progress of rejection [23, 42].

Taken together with the above gene-expression signature which belongs to B lymphocytes, T lymphocytes, and monocytes, it indicates that complex immune responses take place in the donor immune system. Considering their unique role of each immune cell in the enormous immune family, it is not always easy to differentiate acute rejection from chronic one, especially when acute rejection occurs simultaneously with the latter one.

*MAP 4 K1* and *LILRB2* were the two genes upregulated in acute rejection, but not in stable transplant recipients. The data suggest that MAP 4 K1 and LILRB2 probably are the potential checkpoint for the occurrence of acute rejection. MAP 4 K1 plays a role in the activation of c-Jun N-terminal kinase, which contributes importantly to inflammatory responses in mammals [43]. LILRB2 is extensively expressed on immune cells, such as natural killer cells, CD8+ T cells and B cells [44, 45]. By recognising MHC I, LILRB2 prevents lymphocytes from killing MHC I-expressing cells [46]. The increased expression of *LILRB2* indicates an enhanced immune response in acute rejection. However, it is also reported that cytomegaloviruses infection induces spontaneous mutation in MHC I protein and affects the interactions of MHC and LILRB2, suggesting an underlying mechanism of immune escape [14, 47].

In the present study, most DEGs in acute and chronic rejection profiles were enriched in immune responses such as allograft rejection and immune deficiency, implying that the balance between immune defence and attack play a critical role in transplant immunology. Nevertheless, most DEGs from stable recipients were enriched in inflammation, such as chemokine signalling, toll-like receptor signalling, cytokine-cytokine receptor interaction, natural killer cell-mediated cytotoxicity, NF-κB signalling pathway, indicating that inflammation-related-signalling pathways play a role undermining the immune balance [48]. Upregulation of NCF2 in renal biopsies indicates that enhanced oxidative stress is an essential mechanism. Through cleavage by Casp1, interleukin-1 and -18 are secreted from the cell to induce the inflammatory response in neighbouring cells, [49] resulting in intense inflammatory, immune responses and acute rejection [3]. Increased expression of GBP1 is also reported in chronic rejection patients when compared with recipients with stable renal function [15]; however, the involvement of GBP1 in immune responses has not been studied.

*ALB* and *CYP3A4* were the two downregulated genes in the combined comparison of acute rejection groups and stable groups. In clinical research, serum albumin levels are negatively correlated with the outcome of allograft kidney and transplant patients [50–52]. Immunosuppressive agents, including tacrolimus and cyclosporine A, are inactive while bound with proteins. Increased serum concentrations of unbound medicine enhance the efficacy and toxicity of the medication [53]. On the other hand, these immunosuppressive agents cause a reduced synthesis and secretion of albumin in cultured human hepatocytes [54]. Most immunosuppressive medicine, including tacrolimus and cyclosporine A, are substrates of cytochrome P450, [55] but also have inhibitory effects on the enzyme [56]. Cytochrome P450 polymorphisms CYP3A4 in people are extremely high, [57] affecting medicine metabolism and efficacy [58–60].

Other DEGs in the present study, including *TYROBP, ADA, EVI2B, FNBP1,* as well as *GPR65,* have not been thoroughly investigated in inflammation or transplant immunology.

Compared with conventional allograft biopsies, a blood draw of liquid biopsies is less invasive and easier handling [61]. There are several studies profiling renal biopsies and blood biopsies in parallel to monitor dynamic immune changes in transplant patients [13, 37]. Of note, only handful genes are consistently expressed in both peripheral blood and renal biopsies [13, 37], indicating that gene expression profiles of blood are distinctive from those of the biopsies of transplant patients [13]. By combining five public datasets of stable recipients and acute rejection patients, *HIST1H4A* coding basic nuclear protein histone H4 was the only candidate gene upregulated in both peripheral blood samples and renal biopsy [37]. It is reported that the B cell signature genes including *IGKV4–1, IGLL1,* and *IGKV1D-13* are upregulated in tolerant recipients when compared with transplant recipients with stable renal function [23]. However, the upregulation of *IGKV4–1, IGLL1* or *IGKV1D-13* is not reproduced in immune tolerance subjects who were treated with a bioengineered stem cell product [21]. Since no overlapping genes in peripheral blood were identified, there are several interpretations for the inconsistency 1) The activation and recruitment of peripheral blood cells and the subset of lymphocytes to the transplanted kidney are different, regarding sources and underlying mechanisms [13]. 2) The peripheral blood samples are affected by many other factors, including lifestyles, diets, and systemic disease as well as its corresponding therapeutic medicines. 3) Isolation of peripheral blood cells by density gradient purification can activate cells and induce gene changes ex vivo. Thus, it should be cautious of drawing a conclusion on immune status regarding changes in blood samples of transplant recipients.

## Conclusion

Due to the complexity of the immune system, maintaining the balance in immunosuppression, allograft organ rejection, and secondary infection is the ultimate goal for clinicians and organ transplant recipients. By performing bioinformatics analyses on the immune status of renal transplant patients, the present study reports several DEGs in the renal biopsy of transplant recipients, which will be validated in clinical practice.

## Supplementary information

**Additional file 1: Table S1.** GSE datasets of renal transplant patients. **Table S2.** The overlapping DEGs in the comparison of stable and chronic rejection groups. **Table S3.** The overlapping DEGs in the comparison of acute rejection and stable groups. **Table S4.** The overlapping DEGs in the comparison of acute rejection and chronic rejection groups

## Data Availability

GEO datasets recruited in the present study were available on Pubmed/GEO datasets repository. Detailed information listed in the supplemental file (S. Table 1).

## Abbreviations

ADA: Adenosine deaminase
ALB: Albumin
CASP1: Caspase 1
CD: Cluster of differentiation
CYP3A4: Cytochrome P450 3A4
DAP12: DNAX-Activation Protein 12
DEGs: Differentially expressed genes
EVI2B: Ecotropic viral integration site 2B
FCGR3B: Fc Fragment of IgG receptor IIIb
FNBP1: Formin binding protein 1
GBP1: Guanylate binding protein 1,
GEO: Gene Expression Omnibus
GPR65: G protein-coupled receptor 65
HIST1H4A: Histone cluster 1 H4 family member A
HLA: Human leukocyte antigen
IGHG1: Immunoglobulin heavy constant gamma of 1
IGHM: Immunoglobulin heavy constant Mu
IGHV4–-31: Immunoglobulin heavy variable 4–-31
IGKV1D-13: Immunoglobulin kappa variable 1D-13
IGKV4–-1: Immunoglobulin kappa variable 4–-1
IGLL1: Immunoglobulin lambda like polypeptide 1
LILRB: Leukocyte immunoglobulin-like receptor B
LILRB2: Leukocyte immunoglobulin-like receptor B2
MAP 4 K1: Mitogen-activated protein kinase kinase kinase kinase 1
MAPK: Mitogen-activated protein kinase
MHC: Major histocompatibility complex
NCF2: Neutrophil cytosolic factor 2
NF-κB: Nuclear factor kappa B
PSMB9: Proteasome subunit beta type-9
PTPRC: Protein tyrosine phosphatase receptor type C
SRGN: Serglycin
TLR: Toll-like receptor
TYROBP: TYRO protein tyrosine kinase-binding protein
