# Supplementary material for: Bioinformatics analyses on the immune status of renal transplant patients, a systemic research of renal transplantation
Source: BMC Med Genomics. 2020 Feb 11;13:24. doi: 10.1186/s12920-020-0673-6 (PMC7014750; doi:10.1186/s12920-020-0673-6)
Supplement: Supplementary file 1 — Additional file 1: Table S1. GSE datasets of renal transplant patients. Table S2. The overlapping DEGs in the comparison of stable and chronic rejection groups. Table S3. The overlapping DEGs in the comparison of acute rejection and stable groups. Table S4. The overlapping DEGs in the comparison of acute rejection and chronic rejection groups [file 12920_2020_673_MOESM1_ESM.docx]

S Table 1 GSE datasets of renal transplant patients

|  |  |  |
| --- | --- | --- |
| GSE dataset | ID number | Link |
| GSE46474^[7]^ | ID: 200046474 | <https://www.ncbi.nlm.nih.gov/geo/query/acc.cgi?acc=GSE46474> |
| GSE1563^[13]^ | ID: 200001563 | <https://www.ncbi.nlm.nih.gov/geo/query/acc.cgi?acc=GSE1563> |
| GSE51675^[15]^ | ID: 200051675 | <https://www.ncbi.nlm.nih.gov/geo/query/acc.cgi?acc=GSE51675> |
| GSE64261^[15]^ | ID: 200064261 | <https://www.ncbi.nlm.nih.gov/geo/query/acc.cgi?acc=GSE64261> |
| GSE25902^[16]^ | ID: 200025902 | <https://www.ncbi.nlm.nih.gov/geo/query/acc.cgi?acc=GSE25902> |
| GSE9493^[17]^ | ID: 200009493 | <https://www.ncbi.nlm.nih.gov/geo/query/acc.cgi?acc=GSE9493> |
| GSE50058^[18]^ | ID: 200050058 | <https://www.ncbi.nlm.nih.gov/geo/query/acc.cgi?acc=GSE50058> |
| GSE36059^[19]^ | ID: 200036059 | <https://www.ncbi.nlm.nih.gov/geo/query/acc.cgi?acc=GSE36059> |
| GSE98320^[20]^ | ID: 200098320 | <https://www.ncbi.nlm.nih.gov/geo/query/acc.cgi?acc=GSE98320> |
| GSE106675^[21]^ | ID: 200106675 | <https://www.ncbi.nlm.nih.gov/geo/query/acc.cgi?acc=GSE106675> |
| GSE47755^[22]^ | ID: 200047755 | <https://www.ncbi.nlm.nih.gov/geo/query/acc.cgi?acc=GSE47755> |
| GSE22229^[23]^ | ID: 200022229 | <https://www.ncbi.nlm.nih.gov/geo/query/acc.cgi?acc=GSE22229> |
| GSE14346^[24]^ | ID: 200014346 | <https://www.ncbi.nlm.nih.gov/geo/query/acc.cgi?acc=GSE14346> |
| GSE15296^[25]^ | ID: 200015296 | <https://www.ncbi.nlm.nih.gov/geo/query/acc.cgi?acc=GSE15296> |
| GSE66612^[26]^ | ID: 200066612 | <https://www.ncbi.nlm.nih.gov/geo/query/acc.cgi?acc=GSE66612> |

S Table 2 The overlapping DEGs in the comparison of stable and chronic rejection groups

|  | | Stable (Stable vs Healthy) | | Chronic Rejection (Chronic Rejection vs Stable) | | | P-value |
| --- | --- | --- | --- | --- | --- | --- | --- |
| Data Set | | GSE9493 ^[17]^ | GSE1563 ^[13]^ | GSE98320^[20]^ | GSE36059 ^[19]^ | GSE9493 ^[17]^ |  |
| Sample size (n) | | 21 : 15 | 10 : 9 | 326 : 774 | 65 : 281 | 25 : 21 |  |
| DEGs | IGHM | -2.81±1.33 | 14.73±2.37 | 2.40±1.55 | 2.52±1.76 | 2.71±1.68 | 0.637 |
|  | IGHV4-31 | -1.92±0.84 | 3.45±2.34 | 2.43±2.31 | 2.52±2.17 | 6.70±1.34 | 0.927 |
|  | IGHG1 | -2.81±1.23 | -2.97±0.68 | 2.43±2.33 | 2.52±1.71 | 6.70±0.53 | 0.033* |

* P<0.05 vs stable group

S Table 3 The overlapping DEGs in the comparison of acute rejection and stable groups

|  | | Stable (Stable vs Healthy) | | Acute Rejection (Acute Rejection vs Stable) | | | | | | P-value |
| --- | --- | --- | --- | --- | --- | --- | --- | --- | --- | --- |
| Data Set | | GSE9493^[17]^ | GSE1563^[13]^ | GSE106675^[21]^ | GSE9832^[20]^ | GSE36059^[19]^ | GSE25902^[16]^ | GSE1563^[13]^ | GSE50058^[18]^ |  |
| Sample Size (n) | | 21 : 15 | 10 : 9 | 10 : 6 | 81 : 77 | 35 : 281 | 24 : 96 | 6 : 9 | 43 : 58 |  |
| DEGs | ALB | -2.38±1.45 | -2.37±0.57 | -4.11±1.36 | -3.58±2.03 | -1.56±1.74 | -2.50±2.03 | -8.94±0.93 | -2.6±1.53 | 0.446 |
|  | CYP3A4 | 1.44±0.53 | -1.85±0.37 | -1.78±0.40 | -1.46±0.36 | -1.44±0.70 | -1.67±0.72 | -2.53±1.49 | -1.42±0.66 | 0.722 |
|  | CD48 | 1.80±1.00 | 4.08±0.79 | 6.03±0.76 | 5.07±0.63 | 2.68±0.96 | 8.27±1.25 | 4.54±0.92 | 2.75±1.65 | 0.210 |
|  | MAP4K1 | 2.28±0.91 | -1.98±0.48 | 10.63±0.80 | 2.00±0.49 | 1.70±0.98 | 2.30±0.95 | 3.65±0.99 | 2.03±1.25 | 0.254 |
|  | PTPRC | 2.00±1.22 | 2.99±0.67 | 4.90±1.09 | 4.42±0.66 | 3.59±0.99 | 7.39±1.24 | 4.72±1.20 | 3.12±1.25 | 0.072 |
|  | ADA | 2.13±1.01 | 1.74±0.72 | 10.39±0.68 | 2.36±0.44 | 2.01±1.02 | 3.05±0.99 | 5.86±0.48 | 2.24±1.33 | 0.335 |
|  | FNBP1 | 1.50±0.55 | 1.74±0.33 | 1.73±0.53 | 2.26±0.41 | 1.55±0.57 | 1.83±0.63 | 2.75±0.93 | 1.64±0.76 | 0.320 |
|  | EVI2B | 2.17±1.29 | 11.10±1.04 | 2.57±0.93 | 3.50±0.79 | 2.65±0.85 | 7.09±1.13 | 4.98±1.71 | 3.18±1.14 | 0.411 |
|  | LILRB2 | 1.90±1.00 | -2.25±0.68 | 7.29±1.13 | 4.68±0.94 | 3.41±1.28 | 2.11±1.34 | 4.58±1.85 | 1.86±1.21 | 0.052 |
|  | IGHG1 | 2.81±1.04 | -3.13±0.68 | 6.65±1.71 | 4.73±2.10 | 1.49±1.30 | 10.59±2.11 | 2.01±0.71 | 5.72±2.43 | 0.033* |

* P<0.05 vs stable group

S Table 4 The overlapping DEGs in the comparison of acute rejection and chronic rejection groups

|  | | Acute Rejection  (Acute Rejection vs Stable) | | | | | | Chronic Rejection  (Chronic Rejection vs Stable) | | | P-value |
| --- | --- | --- | --- | --- | --- | --- | --- | --- | --- | --- | --- |
| Data Set | | GSE106675  ^[21]^ | GSE98320  ^[20]^ | GSE36059  ^[19]^ | GSE25902  ^[16]^ | GSE1563  ^[13]^ | GSE50058  ^[18]^ | GSE98320  ^[20]^ | GSE36059  ^[19]^ | GSE9493  ^[17]^ |  |
| Sample Size (n) | | 10 : 6 | 81 : 774 | 35 : 281 | 24 : 96 | 6 : 9 | 43 : 58 | 326 : 774 | 65 : 281 | 25 : 21 |  |
| DEGs | TYROBP | 3.20±1.01 | 3.45±0.59 | 2.71±1.02 | 4.33±1.00 | 5.92±0.69 | 3.45±1.31 | 1.82±0.66 | 1.44±0.80 | 2.75±0.77 | 0.040* |
|  | CD86 | 4.07±0.83 | 3.20±0.52 | 2.46±0.90 | 3.28±1.32 | 2.29±0.74 | 3.01±1.11 | 1.64±0.55 | 1.47±0.74 | 1.64±0.88 | 0.006* |
|  | CASP1 | 2.63±0.83 | 2.68±0.41 | 2.18±0.71 | 5.56±0.82 | 3.59±0.79 | 3.28±1.02 | 1.68±0.49 | 1.49±0.65 | 1.58±0.75 | 0.047* |
|  | GPR65 | 3.05±1.08 | 3.76±0.63 | 2.55±0.84 | 3.32±0.89 | 3.58±0.88 | 2.40±1.24 | 1.72±0.69 | 1.46±0.76 | 1.75±0.92 | 0.003* |
|  | NCF2 | 12.60±1.02 | 3.39±0.73 | 2.55±1.00 | 5.48±1.44 | 5.66±1.46 | 2.27±1.64 | 1.82±0.77 | 1.43±0.94 | 1.75±0.92 | 0.156 |
|  | SRGN | 2.66±0.97 | 3.32±0.48 | 2.22±0.67 | 4.87±0.66 | 4.00±1.07 | 2.52±0.81 | 1.70±0.64 | 1.43±0.61 | 1.81±0.43 | 0.033* |
|  | GBP1 | 6.75±1.71 | 4.93±0.96 | 3.73±1.16 | 13.15±0.92 | 4.22±1.17 | 3.59±1.47 | 2.69±0.85 | 1.90±0.90 | 1.82±1.03 | 0.117 |
|  | PSMB9 | 5.55±0.99 | 3.15±0.44 | 2.91±1.02 | 5.97±1.02 | 2.88±0.66 | 2.86±1.75 | 1.87±0.48 | 1.70±0.75 | 1.99±0.95 | 0.053 |
|  | HLA-DPB1 | 4.34±1.25 | 2.33±0.37 | 2.44±0.76 | 4.17±1.02 | 2.95±0.67 | 3.51±1.20 | 1.66±0.46 | 1.53±0.81 | 2.09±0.85 | 0.022* |
|  | HLA-F | 3.13±0.75 | 2.01±0.29 | 1.97±0.71 | 4.80±0.67 | 3.46±0.59 | 1.75±1.54 | 1.44±0.30 | 1.51±0.56 | 1.50±0.64 | 0.093 |
|  | HLA-DPA1 | 3.01±0.76 | 2.90±0.32 | 1.95±0.56 | 3.86±0.60 | 2.17±0.56 | 2.78±1.01 | 2.03±0.58 | 1.53±0.69 | 1.50±0.51 | 0.036* |
|  | HLA-DRA | 4.21±0.63 | 1.73±0.22 | 2.04±0.50 | 4.93±1.87 | 1.66±0.27 | 1.74±1.89 | 1.44±0.32 | 1.48±0.53 | 2.38±0.75 | 0.323 |
|  | HLA-DRB1 | 4.42±0.81 | 2.53±0.37 | 1.95±0.47 | 2.67±0.52 | 2.69±0.55 | 3.67±0.94 | 1.76±0.49 | 1.67±0.44 | 2.66±0.45 | 0.137 |
|  | HLA-DRB4 | 4.42±0.81 | 2.51±0.30 | 3.15±0.49 | 2.81±0.61 | 3.40±0.62 | 3.67±0.94 | 1.76±0.50 | 1.67±0.54 | 2.66±0.76 | 0.025* |
|  | FCGR3B | 7.86±1.67 | 1.83±1.11 | 1.78±0.66 | 6.09±0.81 | 4.81±0.87 | 1.94±1.48 | 2.64±1.05 | 2.18±1.05 | 1.62±0.75 | 0.263 |
|  | IGHG1 | 6.65±1.71 | 4.73±2.10 | 1.49±1.30 | 10.60±2.11 | 2.01±0.71 | 5.72±2.43 | 2.43±2.31 | 2.52±2.17 | 6.70±0.53 | 0.569 |

* P<0.05 vs acute rejection group
